# The Different Tactics of Foot-and-Mouth Disease Virus to Evade Innate Immunity

**DOI:** 10.3389/fmicb.2018.02644

**Published:** 2018-11-12

**Authors:** Gisselle N. Medina, Fayna Díaz-San Segundo, Carolina Stenfeldt, Jonathan Arzt, Teresa de los Santos

**Affiliations:** ^1^Plum Island Animal Disease Center, United States Department of Agriculture, Agricultural Research Service, Orient, NY, United States; ^2^Codagenix Inc., Farmingdale, NY, United States; ^3^Animal and Plant Health Inspection Service, Plum Island Animal Disease Center, United States Department of Agriculture, Orient, NY, United States; ^4^Department of Veterinary Population Medicine, University of Minnesota, St. Paul, MN, United States

**Keywords:** FMDV, evasion innate immunity, apoptosis, autophagy, IFN, cattle/swine, ubiquitin/ISG, virulence factors

## Abstract

Like all pathogens, foot-and-mouth disease virus (FMDV) is recognized by the immune system inducing a heightened immune response mainly mediated by type I and type III IFNs. To overcome the strong antiviral response induced by these cytokines, FMDV has evolved many strategies exploiting each region of its small RNA genome. These include: (a) inhibition of IFN induction at the transcriptional and translational level, (b) inhibition of protein trafficking; (c) blockage of specific post-translational modifications in proteins that regulate innate immune signaling; (d) modulation of autophagy; (e) inhibition of stress granule formation; and (f) *in vivo* modulation of immune cell function. Here, we summarize and discuss FMDV virulence factors and the host immune footprint that characterize infection in cell culture and in the natural hosts.

## Introduction

### The Virus

Foot-and-mouth disease virus (FMDV) is the prototype member of the *Aphthovirus* genus within the *Picornaviridae* family. The virus is the etiologic agent of foot-and-mouth disease (FMD), a disease of cloven-hoofed animals that often causes extensive epizootics in livestock, mostly farmed cattle and swine, although sheep, goats and over 50 wild species can be affected. FMDV exists as seven distinct serotypes: A, Asia-1, C, O and Southern African Territories 1–3 (SAT 1–3), all including numerous subtypes. High morbidity, and broad diversity have made FMD prevention and control challenging.

Similar to other RNA viruses, FMDV bears an error prone polymerase that causes extensive genetic heterogeneity, allowing its existence as viral quasispecies, a phenomenon that permits virus adaptation to rapidly changing environments in the host (Domingo and Perales, 2018). A detailed study of complex virus–host interactions is essential to understand the virus biology and identify potential therapeutic strategies. In this review, we summarize the current knowledge on the strategies FMDV has evolved to evade immune responses in cell culture and in the natural host.

#### Genome Organization

**FIGURE 1:**
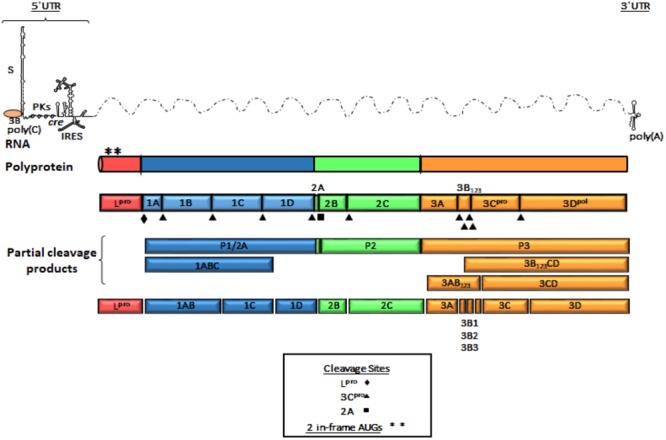
Genome organization of FMDV. A schematic representation of linear (+) stranded FMDV RNA is depicted as thin solid lines and dotted lines represent ORF. Viral genomic RNA contains elements shown at the 5′ and 3′ UTR and are represented as thin lines. The 5′UTR consists of S fragment, poly (C) tract, pseudoknots (PKs), *cis*-acting replicative element (cre) and internal ribosome entry site (IRES). The single ORF encodes a polyprotein and is represented in outlined-open boxes. Filled triangles, squares and diamonds indicate processing sites for 3C^pro^, 2A, and L^pro^, respectively. Post-translational proteolytic cleavages are shown as partial products. Asterisks describe the two AUG initiation codons. The 3′UTR consist of a short stretch of RNA and a poly (A) sequence. The P1 region encodes the structural polypeptides. The P2 and P3 regions encode the non-structural proteins associated with replication. 3B (VPg) protein is shown as covalently linked to the 5′end of the genomic RNA.

The FMDV genome consists of a positive single stranded RNA of approximately 8,500 nucleotides (nt) covalently linked at the 5′end to a viral encoded protein (3B or VPg). The RNA is organized in a relatively extensive 5′UTR, a single ORF and a short 3′UTR (Figure 1). The 5′UTR of about 1,300 nt, is composed of five specific regions that are critical for virus replication and include the S fragment, poly(C) tract, pseudoknots (PKs), *cis* acting replication element (cre) and the IRES (Belsham, 2005). Translation of the ORF begins at two alternative AUG codons, producing a 2,300 amino acid polyprotein that is processed by viral encoded enzymes, resulting in precursors and mature protein products (Vakharia et al., 1987; Clarke and Sangar, 1988; Ryan et al., 1991; Medina et al., 1993; Kirchweger et al., 1994). Mature products include four structural [1A (VP4), 1B (VP2), 1C (VP3), 1D (VP1)] and eight non-structural (NS) proteins [L^pro^, 2A, 2B, 2C, 3A, 3 distinct copies of 3B (VPg), 3C^pro^, and 3D^pol^]. The first mature protein in the ORF is L^pro^, a cysteine protease that self-cleaves from the growing polypeptide chain. L^pro^ is also involved in cleaving many cellular proteins, contributing significantly to virus pathogenesis (Strebel and Beck, 1986; Kirchweger et al., 1994; Brown et al., 1996; Belsham et al., 2000). The P1 region codes for the capsid proteins while P2 and P3 encode for NS proteins that are necessary for viral RNA replication (Gao et al., 2016). 3C^pro^ is a cysteine protease that processes P1, P2, and P3 precursors to generate mature viral products (Vakharia et al., 1987; Bablanian and Grubman, 1993), and 3D^pol^ is the viral RdRp.

#### Virus Life Cycle

The virus cycle of FMDV can be divided in seven distinct phases: binding, internalization, uncoating, translation, replication, encapsidation and cell lysis. FMDV initiates infection by *binding* to integrins via the highly conserved (Arg-Gly-Asp) RGD motif displayed on the surface-exposed G-H loop of VP1 (O’Donnell et al., 2009; Monaghan et al., 2005). In cattle, FMDV mainly binds to αVβ6 integrins highly expressed on epithelial cells (Monaghan et al., 2005; Brown et al., 2006; O’Donnell et al., 2009). However, receptors different from integrins can also be used for entry [i.e., Heparan sulfate (Jackson et al., 1996; Baranowski et al., 2000); Jumonji C-domain containing protein 6 (Lawrence et al., 2016)]. Receptor binding induces *internalization* of FMDV via either CCPs or caveolae–mediated mechanisms (Martín-Acebes et al., 2007; O’Donnell et al., 2008). Following internalization, acidification of the endosomes triggers viral *uncoating* of the icosahedral capsid and genome release into the cytosol (Vázquez-Calvo et al., 2012). *Translation* begins at the IRES element (Belsham, 2009) to produce a polyprotein. FMDV efficiently blocks host translation by an L^pro^-dependent cleavage of the translation initiation factor eIF4G (Devaney et al., 1988; Kirchweger et al., 1994). The FMDV RNA is *replicated* by the viral polymerase 3D^pol^, which along with other FMDV NS products (i.e., 3A, 2B and 2C) concentrates on membranes of the ER and Golgi leading to RNA synthesis (Polatnick and Wool, 1983; Moffat et al., 2005; Midgley et al., 2013). Culmination of the replication cycle requires *encapsidation* of the viral genomic RNA and maturation of the capsid. At this stage the intermediate protein VP0 is processed into VP2 and VP4 by an unknown mechanism (Han et al., 2015). Eventually the infected cell is destroyed (*cell lysis*) causing the egress of newly assembled virus.

### Induction of Innate Immune Responses During Viral Infection

During virus infection, conflicting interests can drive the coevolution of hosts and pathogens: while the host needs to detect the pathogen and stop progression of an infection, usually by mounting a timely inflammatory response, viruses subvert the innate immune system, but avoid overreaction to ensure survival in a live host. In parallel, in the host, the innate sensing is anatomically regulated. Viruses that infect epithelial cells trigger a local IFN response resulting in secreted IFN and other cytokines that induce the expression of ISGs in neighboring and distal tissues, thus increasing hematopoiesis and preparing for systemic viral spread (Hermesh et al., 2010). When the local response fails to restrict virus replication, the virus enters the bloodstream causing robust IFN responses, usually produced by circulating pDCs, (Swiecki and Colonna, 2015). Interestingly, the effectiveness of viral clearance relies upon the particular nature of each virus. While some acute viral infections, resolve in 1–2 weeks, others can become persistent and may reappear when conditions are favorable. Thus, a fine-tuning of the virus–host interplay at the site of infection and systemically in the animal, will faithfully determine the outcome of infection.

#### Innate Immunity Signaling Pathways: Activation

**FIGURE 2:**
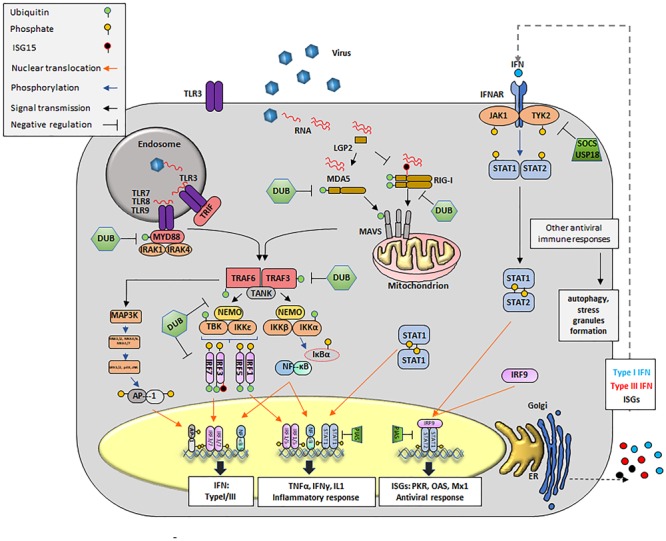
Innate antiviral immune responses during viral infection. Upon viral infection, viral RNA is detected by cytosolic RLRs (RIG-I, MDA-5 or LGP2) and/or membrane bound TLRs, triggering a signal transduction cascade that culminates in the transcription of innate molecules including IFN, pro-inflammatory cytokines and ISGs. Cytosolic RNA is detected by RIG-I, MDA5 and presumably LGP2. RIG-I recognizes RNAs containing 5′ppp and panhandle-like secondary structures. MDA5 recognizes dsRNA. LGP2 might regulate the functions of RIG-I and MDA5. Interaction of RIG-I and MDA5 RNA exposes their CARD domains. RIG-I is subsequently ubiquitinated with unanchored K63-linked Ub chains. MDA5 stacks along dsRNA and form helical filaments. Discrete CARD oligomers (with or without Ub) align on CARD domains of mitochondrial MAVS inducing its polymerization. Endosomal RNAs are detected by TLR3 or TLR7/8, which interact with adaptor proteins TRIF and MyD88, respectively. MyD88 uses other adaptors, IRAK1/4 to allow for interaction with TRAF proteins. Polymerization of MAVS or conformational changes on TRIF and MyD88/IRAK1/4 recruit E3 ligases, mainly TRAF3 and TRAF6. These E3 ligases synthesize poly-Ub that are sensed by NEMO to recruit TBK1 or IKK. NEMO/TBK1 complexes lead mainly to phosphorylation of IRF3/7 (although IRF1 or IRF5 might also be phosphorylated). Phosphorylated IRFs dimerize and translocate to the nucleus. The IKK complex phosphorylates IκB causing its degradation and detachment of NF-κB which then translocates to the nucleus. The same IKK complex can phosphorylate IRF7. Nuclear homo and heterodimers of IRF3 and IRF7 bind to the IFNβ promoter to stimulate its transcription. Nuclear NF-κB binds the IFNβ promoter but also promoters of many proinflammatory cytokines such as TNFα, IL-1, etc. Binding of dimers of phosphorylated IRF7 is essential for the expression of IFNα. In parallel, E3 ligases can activate MAPK3 and subsequently other kinases including ERK1/2 and JNK which phosphorylate the components of the AP1 heterodimer followed by translocation to the nucleus to cooperate with the induction of IFNβ transcription. Secreted IFN can act in an autocrine and paracrine manner by binding to its receptor (IFNR). Upon binding JAKs (JAK1 and TYK2) are activated to phosphorylate the IFNAR receptor, also allowing for JAK dependent phosphorylation of STAT1 and STAT2 (although other STATs may also be affected). Heterodimers of STAT1/2 interact with IRF9 to form the ISGF3G complex which translocate to the nucleus and binds DNA to drive the expression of over 300 ISGs, many of which display antiviral activity. Homodimers of STAT1 translocate to the nucleus and mainly enhance transcription of proinflammatory cytokines. Many proteins of the pathway (RIG-I, MDA5, MAVS, TRAF3/6, MyD88, and IRF3/7/1/5) are targets for deUbiquitination and/or deISGylation and serve as negative regulators of the IFN signaling pathway. For instance, USP18 remove ISG15 from ISG15-conjugated substrates by cleaving the isopeptide bond between ISG15 and its targeted substrate. Other negative regulators of IFN signaling include SOCS and PIAS. Formation of SGs and activation of autophagy can be detected during innate immunity signaling responses.

The primary receptors of innate immunity are composed of a diverse set of PRRs that identify atypical molecules present in viruses, and other microbes, defined as PAMPs, (Kawai and Akira, 2010; Palm et al., 2012; Wu and Chen, 2014) (Figure 2). As PAMPs, viral RNAs are mainly recognized by three types of receptors: endosome associated TLRs, cytosolic RNA helicases known as RIG-I like receptors (RLRs), and NOD-leucine-rich repeat–containing receptors (NLRs). In addition, viral RNA can interact with a family of cellular enzymes such as, dsRNA-dependent PKR, oligoadenylate synthetase proteins (OAS), and others, eliciting a signaling response that limits virus propagation (Kawai and Akira, 2010; Palm et al., 2012; Rathinam et al., 2012; Dempsey and Bowie, 2015; Yoneyama et al., 2015). Of outmost importance in RNA recognition are the TLR and RLR families of proteins (Kawai and Akira, 2010; Wu and Chen, 2014).

#### TLR Activation

Interaction of a TLR with RNA occurs in the extracellular milieu or inside endosomes and within the TLR family, TLR3 recognizes dsRNA (Alexopoulou et al., 2001), while TLR7 and TLR8 sense ssRNA (Heil et al., 2004). Signals sensed through TLRs are transduced through interactions with adaptor proteins including TIR domain proteins (i.e., TRIF, TRAF, etc.), and with MyD88. Successive TLR-driven signals lead to the nuclear translocation of nuclear factor κB (NF-κB, p65/p50) and/or IRF3/IRF7, key transcription factors for IFN and proinflammatory cytokines (Ikushima et al., 2013). In addition, E3 Ub ligases (i.e., TRAF6) can activate the mitogen activated protein kinase 3 (MAPK3) thus inducing the assembly of the AP1, another transcription factor that facilitates IFN mRNA expression (Shaulian and Karin, 2002) (Figure 2).

#### RLR Activation

Three non-membrane bound RLRs have been well characterized: RIG-I, melanoma differentiation-associated gene 5 (MDA5) and LGP2 (Kang et al., 2002; Yoneyama et al., 2004, 2005). RIG-I recognizes relatively short RNA duplexes that contain a tri- or di-phosphate group at the 5′end (Weber et al., 2015; Devarkar et al., 2016) and MDA5 senses longer dsRNA molecules (Kato et al., 2008; Li et al., 2009; Berke and Modis, 2012). At the N-termini, RIG-I and MDA5 proteins contain tandem CARDs that are normally found in a ‘silent signaling’ conformation. Upon interaction with foreign RNA, the CARD domains of these proteins facilitate binding to the MAVS and subsequent aggregation (Seth et al., 2005). In contrast to RIG-I and MDA5, LGP2 is truncated at the 5′end terminus and lacks the CARD domain (Yoneyama et al., 2005). The role of LGP2 in sensing viral RNA is still not completely understood and opposing functions -LGP2 dependent activation and repression- of the IFN pathway have been proposed (Yoneyama et al., 2005; Venkataraman et al., 2007; Satoh et al., 2010). Subsequent events post-CARD active conformation results in the activation of, TBK1, leading to phosphorylation, dimerization and nuclear translocation of IRF3 and IRF7, which strongly induce IFN transcription (Figure 2).

#### IFN Signaling

Downstream from PAMP recognition and ISG induction, IFN autocrine and paracrine loops can perpetuate activation in a calibrated manner. Based on their structural features, receptor usage and biological activities, there are three families of IFNs, types I, II, and III (Donnelly and Kotenko, 2010). As secreted cytokines, IFNs orchestrate a milieu of antiviral responses on infected, neighboring and distal cells including not only virus permissive but also immune cells such as natural killer (NK), APC, and ILCs. IFN signal activation begins extracellularly with the binding to the IFN receptor (i.e., IFNAR1/2), causing the activation of TYK2 and JAK1 which results in the phosphorylation, dimerization and nuclear translocation of the transcription factors signal transducer and activator of transcription 1 (STAT1) and STAT2. STAT1/STAT2 heterodimers can bind to IRF9 to form the complex called the IFN-stimulated gene factor 3 (ISGF3) which in turn binds to DNA to activate the transcription of antiviral genes (i.e., *OAS, PKR, MX1*, etc.) (Figure 2).

#### Innate Immunity Signaling Pathways: Regulation

Many steps of the elaborate IFN induction pathway are tightly regulated, and effectively control viral infection while minimizing autoimmune damage. Of significant importance to these processes are PTMs, i.e., Ub and Ub-like incorporation/removal from several components of the pathogen-sensing and transduction pathways (Heaton et al., 2016). For instance, the ubiquitination and/or deubiquitination of TLR3, TLR9, MyD88, TRIF, TRAF3, TRAF6, Inhibitor of κB kinases (IκB), NEMO, TBK1, IRF3, IRF7, RIG-I, MDA5, MAVS and tripartite motif containing protein 25 (TRIM25) play a critical role in the activation of the key components IRFs, NF-κB, and AP1 (Zhou et al., 2017). In addition, the Ub-like protein (UBL) ISG15, has recently emerged as an important tool in the struggle against many viral pathogens (Skaug and Chen, 2010). Unlike Ub, expression of ISG15 is IFN-induced and can be rapidly upregulated in response to viral infection (dos Santos and Mansur, 2017). ISG15 is linked to target proteins by an isopeptide bond in a mechanism known as ISGylation. This modification has been shown to stimulate and/or block several IFN signaling events (Kim et al., 2008), targeting IRF3, PKR, RIG-I, TANK1, and filamin B among others (dos Santos and Mansur, 2017). In parallel, USP18, another ISG, also affects innate immune responses by removing ISG15 from target proteins thus creating a negative feedback loop that has been reported very important in preventing excessive immune stimulation and autoimmune diseases (Honke et al., 2016). Interestingly, direct interaction between USP18 and IFNAR2 have been shown to contribute in the negative regulation of type I IFN independently of USP18-deISGylase/isopeptidase activity (Malakhova et al., 2006). Other unconventional PTM that may affect the induction of the innate response against RNA viruses have been characterized and are reviewed elsewhere (Zhou et al., 2017).

#### Innate Immunity: Involvement of Other Cellular Processes

Another line of immunity that has been linked with innate responses, is the utilization of preexisting cellular processes and molecules such as those seen during cellular stress: SGs formation, autophagy and apoptosis.

Stress granules are transient non-membrane bound dynamic organelles that store many untranslated mRNPs. SGs usually accumulate upon stress-induced translation arrest and can interfere with viral replication (McCormick and Khaperskyy, 2017). Since, all viruses require the translation machinery of the host to synthesize their own proteins, blockage of SGs represent an strategy to favor their replication. In fact, it has been demonstrated in poliovirus and other picornavirus that formation of SGs is disrupted during infection due to the cleavage of the SG nucleating protein Ras-GAP SH3 domain binding protein 1 (G3BP1) (White et al., 2007; White and Lloyd, 2011; Yang et al., 2018). Interestingly, recent studies have linked virus-induced SGs with RLRs revealing a new function for SGs in sensing viral RNA. Studies by Onomoto et al. (2012) demonstrated that SGs may serve as a platform for RIG-I dependent recognition of viral RNA, at least for influenza viruses. MDA5 has also been detected in SGs (Langereis et al., 2013) suggesting that RLRs can be recruited to SGs and may play an important role in antiviral innate immunity.

Autophagy is an evolutionarily conserved cell-regulated pathway that degrades and recycles long-lived proteins and cellular components. Specifically, under unfavorable conditions such as nutrient deprivation, autophagy mediates a self-eating process via lysosomes. Autophagy-dependent formation of double-membrane vesicles, termed autophagosomes, engulf long-lived proteins, damaged organelles and transport these cargos to the lysosomes where degradation occurs (He and Klionsky, 2009). Much of the orchestration of autophagy, ranging from its induction to vesicular formation and breakdown, are mediated by proteins encoded by ATG (Kuo et al., 2017). Interestingly, signaling pathways that control innate immunity also regulate autophagy (Deretic et al., 2013). For instance, type I IFN enables autophagy (Schmeisser et al., 2014) and upon TLR activation, MyD88 or TRIF can bind to Beclin-1 (ATG9) promoting the maturation of autophagy (Shi and Kehrl, 2008). Additionally, in pDCs, TLR7-dependent production of IFN is impacted upon depletion of the autophagy protein ATG5 during viral infection (Lee et al., 2007). Unexpectedly, autophagy can be beneficial for viruses. For example, poliovirus and rhinovirus are known to usurp the autophagosomal machinery for viral synthesis (Jackson et al., 2005). Some of the reported hijacking strategies include the utilization of autophagosomes as a home for virus replication due to its protected environment and the use of autophagy-generated metabolites and energy required for replication (Choi et al., 2018).

During viral infection, cellular death can occur as a defensive response to limit the production of viruses (Deretic and Levine, 2009). Apoptosis is controlled by internal or external stimulation, both triggering the activation of caspases which leads to cytoskeletal disintegration, metabolic unbalance and culminates with genomic fragmentation. Interestingly, different studies have shown that viruses can use apoptotic processes to produce sufficient virus progeny or to facilitate virus release (Benedict et al., 2002; Ryan et al., 2016). In the case of poliovirus, apoptosis is induced but active infection inhibits its progression (Belov et al., 2003). Undoubtedly, the ultimate outcome following infection will depend on the balance between the competing host and viral influences on the cell death program.

## FMDV Virulence Factors: Evasion of Innate Immunity

Foot-and-mouth disease virus is very effective at replicating in the susceptible host. Exposure to the virus results in close to 100% morbidity. Full disease usually develops by 2–5 days post infection in livestock species such as swine and cattle (Stenfeldt et al., 2014, 2015, 2016a,b). In tissue culture, virus end-point titers can be reached by 4–6 h post infection, depending on the serotype and initial multiplicity of infection. This rapid infectivity relies on many factors, including but not exclusive to, the availability of the specific cellular receptor, the intrinsic viral replication fitness, and the effectiveness of distinct viral proteins to counteract the host innate immune response.

Only a limited number of studies have identified cellular sensors for FMDV infection mostly using cultured primary cells such as porcine/bovine/murine epithelial cells (i.e., derived from kidney, pharynx, lung, and thyroid cells), or myeloid cells (i.e., PBMCs, dendritic cells and macrophages). In addition, many studies have been performed in stable cell lines of porcine/bovine origin such as PK15, SK6, IB-RS2, LF-PK, and MDBK cells, in human HEK293, MCF-10A and HeLa cells or in murine BHK-21 cells. It is noteworthy to mention that when using primary cells, most of the IFN sensing and signaling pathways are intact, mimicking the dynamics of infection in the natural host. In contrast, the knowledge gained from studies conducted in established cell lines may be affected by specific intrinsic cellular abnormalities. For example, commonly used BHK-21 cells only produce limited amounts of IFN upon viral infection (MacDonald et al., 2007). HEK293 cells are deficient in some PRRs such as TLRs and STING (Medvedev and Vogel, 2003; Burdette et al., 2011), although they are able to transduce signals in transient expression experiments using RIG-I, MDA5, TBK1, IRF3, etc. (Dou et al., 2017).

Foot-and-mouth disease virus RNA is recognized by MDA5 but not RIG-I or TLR3 in epithelial porcine PK15 cells (Hüsser et al., 2011). Interestingly a new study revealed that overexpression of the RNA helicase LGP2 can inhibit FMDV replication in these cells, presumably due to a decreased in transcripts involved in inflammatory or anti-inflammatory responses such as CCL3LI, TNF-α, IL-6, IL-4, IL-12, TGF-β1, GM-CSF, and IL-10 (Zhu et al., 2017). However, these observations have not been confirmed in the context of an FMDV infection of the animal host. Nevertheless, these results suggest that LGP2 plays a role in FMDV recognition and support a previous hypothesis proposing a synergistic interaction between LGP2 and MDA5 to mediate antiviral signaling (Bruns and Horvath, 2015). On the other hand, although no studies have been published reporting PKR as a molecular sensor of FMDV RNA, it has been shown that depletion of PKR by gene KO or siRNA, significantly increases virus yield in tissue culture (Chinsangaram et al., 2001; de los Santos et al., 2006). Moreover, expression of PKR mRNA is induced to higher levels after infection with FMDV lacking or containing mutations in L^pro^ in comparison to WT virus (de los Santos et al., 2009). These results suggest that PKR plays a critical role during FMDV infection, presumably not as a RNA sensor, but as an ISG that effectively suppresses host and viral translation upon phosphorylation of eIF2α target (Samuel, 1979).

Besides the limited knowledge on FMDV cellular sensors, it is widely understood that the ability of FMDV to successfully replicate in the host cell, depends on the effective suppression of the induced innate immunity. This activity is contingent upon the expression of L^pro^, the FMDV protein that as defined by Agol and Gmyl, has evolved as a ‘security protein’ to warrant the virus counterattack of the host response (Agol and Gmyl, 2010). Hence, almost every region of the FMDV genome is involved in counteracting the immune response ensuring survival in nature as summarized in Table 1.

**Table 1:** Involvement of structural and non-structural FMDV proteins in modulating/counteracting innate immunity signaling pathways.

FMDV factors	Affected process	Viral counter-mechanism
L^pro^	Translation and transcription	• eIF4G1 cleavage (Devaney et al., 1988; Kirchweger et al., 1994) • Gemin5 cleavage (Piñeiro et al., 2012) • Decreased amounts of IFNβ (de los Santos et al., 2007) • Degradation of NF-κB (de los Santos et al., 2007) • Modulation of PKR (Chinsangaram et al., 2001; de los Santos et al., 2006) • Inhibition of RANTES (de los Santos et al., 2006; Wang et al., 2010) • Induction of ADNP binding to IFNα promoter to disrupt the expression of IFN and ISGs (Medina et al., 2017) • Deubiquitination of proteins involved in innate immunity signaling (RIG-I, TBK1, TRAF3, TRAF6) (Wang et al., 2011a) • DeISGylation (Swatek et al., 2018) • Modulation of IFNβ expression through interaction with LGP2 (Rodríguez Pulido et al., 2018)
2B + 2C and or 2BC	Membrane rearrangements, secretion and trafficking, autophagy and modulation of ISGs expression	• Membrane rearrangements (Monaghan et al., 2004; Teterina et al., 2006) • Inhibition of MHC class I surface expression and secretion of antiviral cytokines (Sanz-Parra et al., 1998; Moffat et al., 2005, 2007) • Modulation of cytopathogenicity (Arias et al., 2010) • Induction of autophagy (O’Donnell et al., 2011; Berryman et al., 2012; Gladue et al., 2012) • Alteration of Ca^2+^ concentrations leading to autophagy (Ao et al., 2015) • Interaction with RIG-I to suppress expression of ISGs and GBP1 (Zhu et al., 2016) • Interaction with LGP2 (Zhu et al., 2017; Rodríguez Pulido et al., 2018) • Induction of apoptosis via interaction with Nmi (Wang J. et al., 2012) • Interaction with IFN-induced protein IF35 (Zheng et al., 2014)
3A	Membranes and innate immunity signaling factors	• Interaction with membranes (González-Magaldi et al., 2014; Lotufo et al., 2018) • Inhibition of RLR (RIG-I, MDA5, MAVS)-mediated IFNβ induction (Li et al., 2016a)
3C^pro^	Transcription, translation and autophagy	• Histone H3 cleavage (Grigera and Tisminetzky, 1984; Falk et al., 1990; Tesar and Marquardt, 1990) • eIF4G and eIF4A cleavage (Belsham et al., 2000) • Sam68 cleavage (Lawrence et al., 2012) • NEMO cleavage (Wang D. et al., 2012) • Reduction of the endogenous levels of PKR (Li et al., 2017) • Interference of JAK-STAT signaling pathway (Du et al., 2014) • Degradation of autophagy proteins ATG5 and ATG12 (Fan et al., 2017) • Cleavage of G3BP1 (SG marker) (Galan et al., 2017; Ye et al., 2018)
VP1, VP2, VP3	Suppression of innate immune signaling responses (type I IFN) and autophagy	• Interaction with the cellular protein sorcin to downregulate transcription of IFNα/β and NF-κB (Li et al., 2013) • Downregulation of TNFα and NF-κB (Ho et al., 2014; Wang et al., 2016) • Induction of autophagy (Sun et al., 2018) • Inhibition of STAT phosphorylation (Li et al., 2016b) • Decrease expression of RIG-I and MDA5 (Li et al., 2016c)
Untranslated regions	Modulation of innate immune signaling	• 5′UTR can stimulate type I IFN responses: Mx-1, IFNβ, IL-6, TNFα, IRF7 (Rodríguez-Pulido et al., 2011; Kloc et al., 2017) • 3′UTR can trigger an antiviral state via IFNβ (Rodríguez-Pulido et al., 2011)

### Leader Protein

Leader (L^pro^) is a papain-like protease that contains a cysteine (Cys)-Histidine (His)-Aspartic acid (Asp) catalytic triad (Guarne et al., 1998). Alike cardiovirus, *Aphthovirus* L^pro^ is encoded at the beginning of the viral ORF, a feature that makes it unique relatively to other picornaviruses. Due to the translation initiation of viral RNA at different AUG codons, two forms of the L^pro^ are expressed, Lab and Lb, which discern by 28 amino acids. These forms free themselves from the nascent viral polyprotein through intra- and intermolecular self-processing events (Steinberger and Skern, 2014). Notably, it has been reported that the physiologically relevant form during viral infection is Lb (Cao et al., 1995), and its expression is abundantly observed *in vitro*. L^pro^ induces cleavage of the translation initiation factor eIF4G, including eIF4GI and eIF4GII (Devaney et al., 1988; Medina et al., 1993; Gradi et al., 2004), resulting in the inhibition of cellular cap-dependent protein synthesis. In addition, phosphorylation of eIF2α, as a response to stress, also contributes to the strong translation arrest induced by the virus infection. However, protein synthesis on the FMDV RNA is maintained due to its dependence on the IRES contained within the 5′-UTR (Belsham, 2009), and the L^pro^ ability to enhance IRES-driven translation in the presence of phosphorylated eIF2α (Moral-López et al., 2014).

During FMDV infection, L^pro^ can drive many specific countermeasures to overcome the host innate immune defenses. In addition to its primary function to broadly and efficiently inhibit translation of all host capped mRNAs, including molecules involved in innate and adaptive immunity, FMDV L^pro^ causes degradation of p65/RelA, a subunit of the transcription factor NF-κB, thus blocking its activity in modulating pro-inflammatory cytokine expression (de los Santos et al., 2007). Degradation of this NF-κB subunit not only requires catalytic activity, but also an intact SAP domain on L^pro^ (de los Santos et al., 2009). Interestingly, mutations in the SAP domain prevented nuclear retention of L^pro^ and degradation of NF-κB (de los Santos et al., 2009). Subsequent studies demonstrated that L^pro^ significantly inhibits NF-κB-dependent gene expression, including IFNβ and many ISGs during infection (Zhu et al., 2010). In overexpression studies in PK15 cells, L^pro^ can also decrease the IRF-induced IFNα/β expression by reducing IRF3 and IRF7 protein levels independently of its protease catalytic activity. However, the specific L^pro^ dependent mechanism that regulate IRF3 and IRF7 protein turnover remains undetermined (Wang et al., 2010). In contrast, decrease in the levels of IFNλ1 transcripts in cultured cells overexpressing L^pro^ requires an intact L^pro^ catalytic activity (Wang et al., 2011b). These results suggest that the specific determinants of L^pro^ virulence are not completely understood. Studies directed to examine L^pro^ cellular protein target affinity and/or the spatial regulation during FMDV infection may provide additional clues.

Many of the approaches evolved by FMDV L^pro^, are directed to hinder the connection between transcription factors and IFN promoters. In fact, L^pro^ can also regulate the host transcriptional machinery by directly binding the transcription factor ADNP (activity-dependent neuroprotective protein). Specifically, during infection, WT FMDV but not LLV (Piccone et al., 1995), induced ADNP binding to IFN-α promoter disrupting the expression of IFN and ISGs (Medina et al., 2017). Furthermore, L^pro^-ADNP complex was found in association with chromatin remodeling protein Brg-1 indicating a potential interplay between FMDV and the epigenetics machinery that modulates the antiviral response. Targeting specific epigenetic mechanisms that influence expression of TNFα, NF-κB1a, IFNβ, and IL-12b and IL-6 provides a potential advantage during virus infection (Ramirez-Carrozzi et al., 2009). In fact, in a recent study, the role of such influences during FMDV infection has been examined. Specifically, blocking of the EHMT2 during FMDV infection resulted in the upregulation of IFNβ, ISG15, Mx-1, Mx-2, RIG-I, OAS-1, and PKR transcripts in bovine cells, and led to a significant reduction in virus replication (Singh et al., 2016).

As mentioned above, it has been reported that during infection, FMDV induces transcription of LGP2 mRNA but limits its protein expression (Zhu et al., 2017). Interestingly these authors found that overexpression of NS viral proteins, 2B, L^pro^ and 3C^pro^, were responsible for this function, independently of their intrinsic proteolytic activity. More recently Rodríguez Pulido et al. (2018) demonstrated that L^pro^ directly cleaves LGP2 resulting in reduced IFNβ mRNA expression. Specific cell type used in these analyses may explain these differences. Notwithstanding, both studies showed that overexpression of LGP2 resulted in a significant reduction in FMDV replication.

Foot-and-mouth disease virus L^pro^ has also been implicated in removing Ub molecules from several immune signaling molecules including RIG-I, TBK1, TRAF3, and TRAF6 (Wang et al., 2011a), thus inactivating downstream signaling. In elegant experiments, Swatek et al. (2018) have recently shown that FMDV L^pro^ is able to remove ISG15 from cellular proteins *in vitro* on synthetic substrates and, on cellular targets during virus infection. Although evidence for L^pro^ ability to deISGylate innate immune signaling proteins has thus far, not been shown, the peculiar disengagement of ISG15 from substrates upon FMDV L^pro^ targeting suggests that L^pro^ could prevent ISG15 recycling and thus affect many host responses.

### 3C^pro^

Foot-and-mouth disease virus 3C (3C^pro^) is a protease responsible for the proteolytic cleavage of most of the viral polypeptide into the functional proteins required for virus replication (Vakharia et al., 1987; Clarke and Sangar, 1988). 3C^pro^ is a chymotrypsin-like cysteine protease (Birtley et al., 2005) that has been associated with inhibition of host cell transcription and translation. While L^pro^ is responsible for cleavage of eIF4G resulting in the hallmark shutoff of host protein synthesis during FMDV infection, a role for 3C^pro^ in this process has also been reported. 3C^pro^ can cleave eIF4G and the cap-binding complex eIF4A although these events take place at later times post infection (Belsham et al., 2000). In the case of cellular transcription interruption, 3C^pro^ induces the cleavage of histone H3 during FMDV infection (Grigera and Tisminetzky, 1984; Falk et al., 1990; Tesar and Marquardt, 1990).

Targeting of 3C^pro^ to immune signaling pathways has been recently reported and include the direct cleavage of NEMO, which bridges the activation of NF-κB and IRF signaling pathways (Wang D. et al., 2012). It has been recently reported that 3C^pro^ also mediates the direct cleavage of Sam68, a component of SGs related to the host stress/antiviral response (Lawrence et al., 2012). Furthermore, Ye et al. have showed that during FMDV infection 3C^pro^ cleaves G3BP1, another SG marker (Ye et al., 2018). It has been reported that G3BP1 also binds to FMDV IRES hindering viral translation (Galan et al., 2017). Cleavage of G3BP1/2 may favor viral infection not only by preventing SG modulation of host antiviral responses but also by eliminating a negative function on IRES dependent viral translation.

Foot-and-mouth disease virus 3C^pro^ has been also associated to degradation of other protein host factors, however, no direct targeting has been so far demonstrated. A decrease of endogenous PKR have been correlated to overexpression of FMDV 3C^pro^, but protein processing was mediated by lysosomal degradation (Li et al., 2017). In addition, interference of the JAK/STAT signaling pathway has been observed in IFNβ treated HeLa cells overexpressing 3C^pro^. Specifically, 3C^pro^ suppressed the ISRE promoter activities and inhibited the nuclear translocation of STAT1/STAT2 heterodimers due to the degradation of KPNA1 (Du et al., 2014). More recently, a study has reported the targeting of proteins involved in autophagy by FMDV 3C^pro^. In the context of FMDV infection, autophagy seems to be beneficial for infection (O’Donnell et al., 2011; Gladue et al., 2012), especially during virus entry (Berryman et al., 2012). However, autophagy-associated antiviral responses can be elicited during virus infection (Xu and Eissa, 2010). To countermeasure these responses, FMDV 3C^pro^ can stimulate the degradation of autophagy proteins ATG5 and ATG12 negatively regulating the NF-κB pathway, the phosphorylation of TBK1 and the activation of IRF3 (Fan et al., 2017).

### 2B

The non-structural protein 2B is known to be involved in the rearrangement of host cell membranes and disruption of the cellular secretory pathways (Moffat et al., 2005, 2007), a function that may be further enhanced by the viral 3C^pro^ which causes Golgi fragmentation (Zhou et al., 2013). FMDV 2B is considered one of the most conserved regions in the entire FMDV genome (Carrillo et al., 2005) and molecular modeling analysis have suggested that it has similar features of a viroporin (Nieva et al., 2012). Specifically, FMDV 2B contains two transmembrane domains and localizes primarily in the ER (Moffat et al., 2007). Nearly alike to known viroporins, 2B oligomerizes to form homomultimers and is involved in membrane rearrangements that are required for efficient virus replication. It is thought that these membrane alterations contribute to the formation of intracellular niches that prevent detection of the virus just like the induction of autophagosomes during infection. In addition, FMDV 2B has been shown to effectively change Ca^2+^ concentrations in the cytoplasm and consequently stimulate autophagy (Ao et al., 2015). Notably, during FMDV infection, 2B can be found in association with autophagosomes markers (O’Donnell et al., 2011).

Direct connection between FMDV 2B and antiviral response mechanisms have recently become evident. *In vitro* experiments have identified a direct interaction between FMDV 2B and RIG-I which induced the reduction of RIG-I expression in porcine PK-15 cells (Zhu et al., 2016), a secondary porcine kidney cell line normally used to examined IFN stimulation and IFN-inducible genes due to the presence of an intact IFN signaling pathway (Chinsangaram et al., 2001; de los Santos et al., 2006). Examination of the RIG-I mediated signaling transduction events indicated that PK-15 cells overexpressing FMDV 2B suppressed ISG15 and IFN-induced GBP1, adding a novel mechanism to counteract antiviral responses. Interestingly, FMDV infection sparked an increase in LGP2 transcripts while significantly reducing LGP2 protein abundance in porcine PK-15 cells. Positive and negative regulatory functions on IFN and inflammatory cytokines have been ascribed to LGP2 during a viral infection (Satoh et al., 2010; Si-Tahar et al., 2014). Consistent with these results in the context of FMDV infection, LGP2 can function as a suppressor of expression of TNFα, IL-6 IL-4, and CCL3L1 (Zhu et al., 2017), or as an inducer of IFNβ (Rodríguez Pulido et al., 2018). As mentioned above, similarly to L^pro^ and 3C^pro^, FMDV 2B associates to LGP2 upon co-expression in HEK293T cells (Zhu et al., 2017).

Synergistic functions between FMDV 2B and 2C (Moffat et al., 2007) have been reported and together can interrupt protein secretions that could affect the transport of major histocompatibility class (MHC) molecules by blocking ER-to Golgi traffic (Moffat et al., 2007). Alterations in MHC-I cell surface expression have been previously reported in epithelial cells infected with FMDV (Sanz-Parra et al., 1998) indicating a mechanism that dodges FMDV detection by preventing the formation of MHC-I peptide complexes on the plasma membrane.

### 2C

The non-structural protein 2C is the largest membrane-binding component of the virus and contains a predicted amphipathic helix domain at its N-terminus (Teterina et al., 2006) which is thought to be required for membrane rearrangements (Monaghan et al., 2004). Given its capacity to enhance membrane alterations, bind to ssRNA and display ATPase activity (Sweeney et al., 2010), 2C acts as an important factor in FMDV viral replication. FMDV 2C has been associated with autophagy as immunofluorescence microscopy experiments indicated the colocalization between FMDV 2C and autophagy markers (O’Donnell et al., 2011). In addition, FMDV 2C can interact with the host protein Beclin-1, a well-known key regulator of autophagy that promotes FMDV replication by inhibiting the fusion of lysosomes with autophagosomes (Gladue et al., 2012). Another aspect of FMDV 2C function involves the interaction with N-myc and STAT (Nmi), a cellular protein known to interact with STATs to augment STAT-mediated transcription in response to cytokines such as IL-2 and IFNγ and can be found in the mitochondria and ER (Wang J. et al., 2012). Interestingly, such interaction resulted in the induction of apoptosis, as defined by the presence of activated caspase-3 and DNA fragmentation markers in BHK-21 cells, albeit not in the context of FMDV infection. Although, induction of apoptosis by FMDV is somewhat controversial and may be cell-dependent, additional examination of this cellular pathway is warranted. Furthermore, co-immunoprecipitation assays and colocalization detected by confocal microscopy, indicated the association of FMDV 2C with the IFN-induced protein 35 (IFP35) (Zheng et al., 2014), a factor with roles in antiviral and cytokine responses. Interestingly, IFP35 have been found to negatively impact the activation of RIG-I favoring virus infection (Das et al., 2014), however, overexpression of FMDV 2C in HEK293T cells resulted in Nmi-induced activation of type I IFN promoters which was found to be dependent on the expression of IFP35 (Zheng et al., 2014) since its depletion obliterated the response.

### 3A

Among all picornaviral 3A proteins, FMDV 3A is the largest, containing 153 amino acids as compared to only 89 amino acids for poliovirus 3A (Mason et al., 2003). Deletions and point mutations in 3A have been associated to altered host specificity, adaptation, attenuation and virulence. For instance, a deletion in the C-terminus region of 3A was found in a strain of FMDV that caused the 1997 outbreak in Taiwan and exclusively affected swine (Dunn and Donaldson, 1997). Interestingly, FMDV isolates from pigs carrying deletions in 3A grew up well in porcine cells and caused disease in swine but displayed restricted growth in bovine cells *in vitro*, and only signs of subclinical FMD in bovines in *in vivo* experiments (Pacheco et al., 2013; Stenfeldt et al., 2018). Furthermore, a single mutation in 3A provided adaptation of FMDV to guinea pigs suggesting this protein participates in determining host range specificity (Nunez et al., 2001). Examination of FMDV 3A membrane topology has revealed a hydrophobic domain that facilitates its interaction with cellular membranes (González-Magaldi et al., 2014). This domain has been detected partially in association with ER and Golgi markers (O’Donnell et al., 2001; Garcia Briones et al., 2006), being the ER a critical organelle for FMDV replication (Midgley et al., 2013). In addition, functionality assays using a DNA-launched luciferase reporter replicon system for FMDV, reported that mutations in FMDV 3A hydrophobic domain resulted in reduced viral replication and nuclear translocation in BHK-21 cells (Lotufo et al., 2018). Recent data points to the involvement of FMDV 3A and the inhibition of RLR-mediated IFNβ induction (Li et al., 2016a). In this study, overexpression of FMDV 3A in HEK293 cells resulted in decreased transcript expression of RIG-I and MDA5, and inhibited SeV-induced activation of IRF3. Interestingly, co-immunoprecipitation experiments indicated that an overall hydrophobic region comprising the first 102 amino acids of 3A are required for association with RIG-I, MDA5, and MAVS. Notably, RIG-I and MAVS associate with mitochondrial-associated membranes (MAM) which can establish innate immune synapses with the ER and can be targeted by viral proteases to ablate RIG-I signaling (Horner et al., 2011). Thus, targeting of these membrane-complexes by FMDV 3A may prevent the effective organization of RLR-signaling pathways.

### Structural Proteins: VP1, VP2, and VP3

Reports on the antagonism of structural proteins are limited, since modification of the cellular landscape is mainly accomplished through the actions of the above-mentioned non-structural proteins, i.e., L^pro^, 3C^pro^, etc. However, recent evidence engaged FMDV structural proteins VP1 and VP3 in the suppression of innate immune signaling responses that are primarily driven by type I IFN. A yeast two-hybrid screen using a swine spleen cDNA library identified the interaction between VP1 and the cellular protein sorcin (soluble resistance-related calcium binding protein), a protein that seems to regulate cell response to viral infections (Li et al., 2013). The functionality of this interaction was examined in HEK293 cells by overexpression of VP1 and indicated a reduction on the TNFα and (SeV)-induced activation of IFNα/β and NF-κB transcription. Interestingly, mouse PMCs treated with recombinant FMDV VP1–VP4 proteins resulted in the downregulation of TNFα and other cytokines, while expression levels of CCL19, IL-15, IL-9, GM-CSF, and Galectin-1 were significantly upregulated (Wang et al., 2016). Furthermore, downregulation of IKK/NF-κB has been observed in human lung cancer cells treated with recombinant FMDV VP1 (Ho et al., 2014) indicating a direct relationship between VP1 and the regulation of innate immunity.

Most recently, a report has highlighted the biological role of FMDV VP2 in the induction of autophagy (Sun et al., 2018). In this study, transfection of porcine cells PK-15 with FMDV VP2 resulted in the activation of eIF2α-ATF4 pathway, which plays a key role in regulating the autophagy gene transcription program in response to stress (B’Chir et al., 2013). In addition, activation of this pathway was dependent on the interaction between FMDV VP2 and the HSPB1. Interestingly, mutations known to affect antigenicity and pathogenicity of FMDV also blocked FMDV VP2 association with HSPB1 leading to the inhibition of autophagy (Xue et al., 2012).

In the case of VP3, direct antiviral activity was reported in overexpression studies using HEK293T cells which resulted in the inhibition of phosphorylation-mediated regulation of STAT, and the blockage of the JAK1/STAT1 complex (Li et al., 2016b). In this study, FMDV VP3 was found in association with JAK1 which affected JAK1 protein levels by promoting degradation through the lysosomal pathway. In addition, FMDV VP3 significantly inhibited SeV-triggered activation of the IFNβ promoter leading to the decrease in transcription of IFNβ, CXCL-10, ISG56, and RANTES. Furthermore, FMDV VP3 has been found to block IRF3 phosphorylation and dimerization and decrease the expression of RIG-I and MDA5 (Li et al., 2016c). Interestingly, co-immunoprecipitation studies showed that FMDV VP3 interacts with MAVS and their association was dependent on the presence of the transmembrane domain in MAVS and the C-terminal domain in VP3.

Overall, the recent discoveries on the involvement of FMDV structural proteins beyond their well-defined role in virus assembly and antigenicity in the animal host, suggest a novel function to modulate innate immunity signaling pathways during internalization and replication. It has been reported that during picornavirus infection molecular chaperones (i.e., Hsp90) alleviate competing constraints determined by protein stability, propensity to aggregation and translation kinetics (Geller et al., 2018). Furthermore, it has been proposed that chaperones interact with PRRs to ensure proper folding and affect innate immune signaling (Binder, 2014). It is possible that during FMDV infection, chaperones assist for the proper assembly of capsid proteins and consequently affect innate immunity signaling. In fact, it was recently demonstrated that Hsp90 is required for FMDV capsid precursor processing and pentamer assembly (Newman et al., 2017). These results further support a role of FMDV structural proteins in modulating the innate immune response.

### Untranslated Regions

The 5′ UTR of FMDV is over 1,300 bases in length and exhibits the most intricate organization among picornaviruses, comprising the S region, a poly(C) tract, several pseudoknots, the *cis*-acting replication element (*cre*) and the IRES, all involved in many facets of replication (Kühn et al., 1990; Martínez-Salas et al., 2015; Kloc et al., 2018). Recent studies have also implicated the FMDV 5′ UTR in modulation of innate immune signaling pathways. For instance, *in vitro* transcribed FMDV non-coding regions transfected in SK6 porcine cells or injected into mice triggered immune responses mediated by type I IFN and reduced susceptibility against FMDV (Rodríguez-Pulido et al., 2011). This antiviral cellular state has also been observed in a study targeted to identify the minimal FMDV S fragment sequence required to stimulate IFNβ mediated pathways (Kloc et al., 2017). Specifically, a genetically modified FMDV containing deletions in the S fragment resulted in attenuation in primary bovine kidney cells and in mice. Furthermore, examination of IFN and ISGs mRNA transcripts in cells infected with FMDV S fragment mutant demonstrated an upregulation of Mx-1, IFNβ, IL-6, TNFα, and IRF7 when compared to WT virus infection. Whether or not the establishment of a defined FMDV 5′ UTR fragment-stimulated antiviral response is linked to specific interactions with host proteins, remains to be determined. However, many host cellular proteins have been found to interact with the 5′UTR. Among them, the cellular factors PTBP, IRES transacting factor (ITAF45), PCBP2 and nucleolin, support viral translation or RNA stability (Luz and Beck, 1991; Pilipenko et al., 2000; Andreev et al., 2007). These proteins regulate IRES activity providing cell type specificity and determining virus spread, and some of them are cleaved during infection (Rodríguez Pulido et al., 2007). Other proteins, such as Gemin5, downregulate IRES driven translation, but this effect is neutralized during FMDV infection, since this protein is cleaved by L^pro^ (Pacheco et al., 2009; Piñeiro et al., 2012). Another host factor that interacts with the 5′ UTR is RHA, a cellular protein that binds the S fragment and the viral NS proteins 2C and 3A. During FMDV infection, RHA, a methylated nuclear protein, is re-localized in a non-methylated form to the cytoplasm of the cell favoring viral replication (Lawrence and Rieder, 2009; Lawrence et al., 2014).

The 3′UTR FMDV comprises a structural sequence of 90 nt folding into two separate stem-loops and a poly (A) tail with variable lengths (Belsham, 2005; Serrano et al., 2006), both involved in viral replication and virulence (García-Nuñez et al., 2014). As displayed by other picornaviruses, FMDV show long-distance interactions between both terminal ends of the genomic RNA to support coordination of viral protein and RNA synthesis (Serrano et al., 2006). Interestingly, construction of a virus containing a deletion in the 3′UTR resulted in a non-viable virus (Sáiz et al., 2001) indicating that this region is critical for FMDV infectivity and replication. Interaction between FMDV 3′UTR and the innate immune response has been reported and is linked to type I IFN. Specifically, examination of IFNβ expression in SK6 porcine cells transfected with FMDV 3′UTR transcripts resulted in an effectual response, which was also confirmed *in vivo* (Rodríguez-Pulido et al., 2011). Importantly, deletions within the secondary structures of FMDV 3′UTR negatively impacted the stimulation of an antiviral cellular state, presumably because RNA structure conservation in these regions may be important for recognition by PRRs.

## Fmdv Pathogenesis in Cattle and Swine

The viral and host components of the pathogenesis of FMDV infection have been described in detail in cattle and pigs, delineating commonalities and differences across species. Defined pathogenesis events have guided research into innate immunity on the basis that the host responses are expected to originate at sites of infection and subsequently continue with systemic cascades affecting distant target tissues. In cattle, primary FMDV infection has been localized to distinct regions of lymphoid-associated epithelium of the nasopharyngeal mucosa (Arzt et al., 2010; Stenfeldt et al., 2015). In cattle that are experimentally exposed to aerosolized virus, primary infection of the nasopharynx is followed by a phase of viral amplification in the lungs (Brown et al., 1996; Arzt et al., 2010; Pacheco et al., 2010a). However, this distinct phase of FMDV pathogenesis does not occur in cattle that have been infected through intra-nasopharyngeal inoculation or natural contact exposure (Stenfeldt et al., 2015). The subsequent clinical phase of disease involves systemic generalization and virus amplification in vesicular lesions at peripheral sites, including the oral mucosa and coronary bands of the feet. FMDV pathogenesis in cattle is further complicated by the occurrence of a prolonged subclinical persistent phase of infection, which does not occur in pigs (Stenfeldt et al., 2016b). During this FMDV carrier state, which occurs in approximately 50% of infected cattle, infectious FMDV is similarly restricted to distinct regions of lymphoid-associated epithelium of the nasopharyngeal mucosa, as occurs during primary infection (Zhang and Kitching, 2001; Pacheco et al., 2015; Stenfeldt et al., 2016a).

In pigs, primary FMDV infection has instead been demonstrated to occur within epithelial crypts of the oropharyngeal tonsils (Stenfeldt et al., 2014). This anatomic difference in primary FMDV infection between cattle and pigs is consistent with an apparent difference in susceptibility to infection via inhalation versus oral exposure (Donaldson et al., 1987; Donaldson and Alexandersen, 2001; Alexandersen and Donaldson, 2002). However, despite the different anatomic location, the micro-anatomic and phenotypic characteristics of the distinct regions of lymphoid-associated epithelium that support primary FMDV infection are highly similar in both host species.

### FMDV Driven IFN Response *in vivo*

There is a strong consensus amongst published works indicating the occurrence of a substantial activation of systemic type I and/or type III IFN activity concurrent with the onset of viremia during the fulminant, acute clinical phase in unvaccinated FMDV-infected cattle (Stenfeldt et al., 2011; Windsor et al., 2011; Perez-Martin et al., 2012; Arzt et al., 2014; Eschbaumer et al., 2016). This innate response has been demonstrated with variable detection of bio-active IFN through reporter assays and/or induction of mRNAs for IFN and ISGs in PBMCs (Perez-Martin et al., 2012). The correlation between the appearance of high quantities of virus in the blood, and upregulated systemic antiviral activation is supported by the finding that vaccinated cattle that are protected from generalization of infection, typically do not have viremia, and similarly lack the systemic IFN activation that occurs in naïve cattle (Eschbaumer et al., 2016). However, thus far it remains unclear, which is the source of systemic IFN in cattle. Two distinct mechanisms have been proposed based on experimental data. Bovine pDCs have been implicated through *ex vivo* studies, as a potential source of the high levels of IFN in response to FMDV immune complexes (Reid et al., 2011). This finding is further supported by the demonstration of up-regulation of IFNβ and λ3, and ISG [e.g., Mx-1, OAS-1, CXCL10, ISG15, OAS1, and RIG-I] mRNAs in PBMCs concurrent with establishment of viremia (Perez-Martin et al., 2012). Alternatively, several studies have demonstrated significant induction of inflammatory and antiviral factors at sites of lesions with abundant viral amplification (Zhang et al., 2009; Arzt et al., 2014; Stenfeldt et al., 2018). These sites include the characteristic vesicular lesions that develop on the tongue, within and around the oral cavity, as well as in coronary band- and interdigital cleft epithelium during the clinical phase of disease. The finding of extremely high induction of IFNα (>1000-fold) and IFNλ (>2400-fold) mRNAs at lesion sites suggests that innate mediators produced at sites of high levels of viral replication may enter the systemic circulation and thereby induce innate responses at distant sites (Arzt et al., 2014).

There are fewer published records and, in some instances controversial results, characterizing the systemic IFN response to FMDV infection in pigs. Nfon et al measured IFNα protein in porcine serum following infection with FMDV. Interestingly concomitant with the onset of viremia, different IFNα amounts were detected depending on the specific FMDV serotype used for infection (Nfon et al., 2010; Summerfield, 2012). However, only few animals were used for the study, no statistical significance could be determined and the levels of systemic IFN were lower than those detected in cattle upon FMDV infection. A separate study found that there was no detectable induction of systemic IFNα in naïve pigs following challenge with FMDV A24 (Diaz-San Segundo et al., 2010). Further studies are required to determine whether or not, FMDV interferes with the induction of systemic IFN in the swine.

A number of studies have investigated the role of local antiviral response activation at the site of primary infection in the bovine nasopharyngeal mucosa. However, the evidence of an activated innate response in the mucosal tissue is not as consistent as the strong systemic antiviral response detected in the host species (Arzt et al., 2010; Windsor et al., 2011; Stenfeldt et al., 2015). This may be due to the substantially lower level of FMDV replication that occurs at the sites of primary infection as compared to the massive viral amplification that occurs in vesicular lesions in the tongue and the feet. Some investigations have reported a low to moderate upregulation of ISGs, including OAS and Mx-1, in the nasopharyngeal mucosa concurrent with establishment of viremia (Arzt et al., 2014; Stenfeldt et al., 2018). Interestingly, these same publications reported non-significant down-regulation of IFNα and β mRNA in nasopharyngeal tissue samples harvested at 24, 48, and 72 h post aerosol inoculation (hpi), whereas IFNλ mRNA was variable, up- or down- regulated. It is known that IRF7, is the master regulator of IFNα expression and strong upregulation is detected upon viral infection (Honda et al., 2005); however, no consistency in the expression of IRF7 could be detected in bovine tissues isolated from FMDV infected cattle, with up- or down- temporal regulation depending on the study (Stenfeldt et al., 2018). A different study showed upregulation of IFNα, β, γ, and λ mRNA in distinct micro-anatomic compartments of the nasopharyngeal mucosa concurrent with occurrence of viremia in non-vaccinated cattle (Stenfeldt et al., 2015). Interestingly, this upregulation of local antiviral activity was more pronounced, and occurred earlier in cattle that had been vaccinated, and in which FMDV infection was restricted to the nasopharyngeal mucosa (Stenfeldt et al., 2015). Consistent with transcriptomic evidence of activation of inflammatory or antiviral responses at the site of primary infection, investigations by immuno-microscopy have demonstrated recruitment of CD 11c/major histocompatibility complex II (CD11c+/MHCII+) cells (presumptively DCs) to distinct focal regions of FMDV-infected epithelial cells within the nasopharyngeal mucosa at 24–72 hpi (Arzt et al., 2010; Stenfeldt et al., 2015). The identification of these cells at early times after infection suggests mechanisms that are driven by innate immunity mediators. However, similar to regulation of antiviral genes, this distinct influx of APCs was more consistent, and occurred at an earlier stage of infection in cattle that had been vaccinated prior to virus exposure (Stenfeldt et al., 2015), suggesting that priming of adaptive immune processes may enhance the response.

Less work has been published on the characterization of the innate response to FMDV infection in porcine tissues. One investigation found that while the levels of INDO, MIP3α, and MCP-1 mRNAs are induced in skin early post infection, no consistent change could be detected in the pattern of pro-inflammatory cytokines (IL-12, -15, or -18) expression (Diaz-San Segundo et al., 2010).

Overall, these data suggest that cattle generate a robust IFN response during the fulminant phase of FMD in the presence of abundant quantities of virus and viral RNA. However, the mechanisms and magnitudes of innate responses at the primary infection site are less clear, with contrasting evidence for activation and inhibition of innate immune processes. The findings from *in vivo* investigations in cattle suggest that there is a relationship between the magnitude of FMDV replication, and activation of the innate immune response. Only limited information is available regarding the local and systemic innate response to FMDV infection in pigs. Further research in this field is warranted as new technologies and reagents in immunology become available.

### FMDV Modulates Early Stages of Cellular Immunity *in vivo*

As mentioned above, the FMDV infectious cycle in individual animals is short. The virus infects, replicates, spreads throughout the body and is shed in less than 7 days. Evidently, FMDV accomplishes such a rapid colonization of the host by manipulating the early innate immune response creating a window of opportunity that allows dissemination prior to the establishment of adaptive immunity. Understanding of the host-pathogen interaction and viral escape mechanisms of immunity is particularly important in cattle and swine because they represent one of the most important livestock industries worldwide and in swine, acutely infected pigs shed substantial quantities of virus into the environment. Furthermore, high scale production of swine has gained momentum in recent years with the development and increased demand in many countries of East Asia, particularly China and South Korea.

**FIGURE 3:**
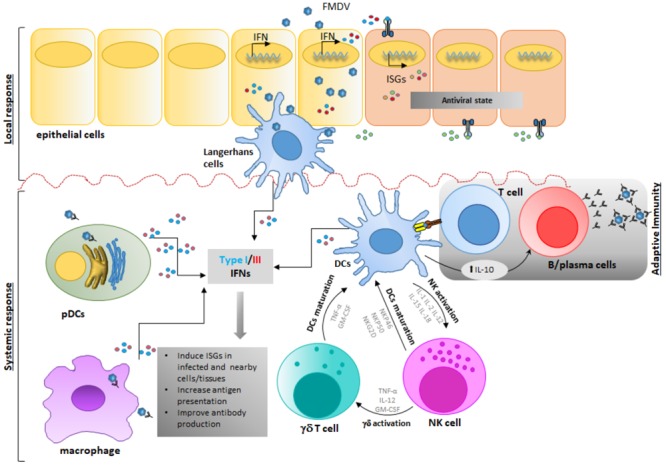
Foot-and-mouth disease virus modulates early stages of immune response *in vivo*. Upon FMDV infection and first round of replication in epithelial cells-the primary infection site-, FMDV gets in contact with cells of the innate immune response inducing functional consequences that affect the host response. FMDV interacts with different players of the immune response either as a result of lytic infection of epithelial cells and subsequent phagocytosis, and/or lytic action on damaged infected tissue or by direct infection of immune cells through a direct or an antibody-dependent internalization process in Mφ, DCs, or NK or γδ T cells. After this stage, DCs, pDCs and Mφ produce IFN and other cytokines that modulate the immune response. Possible interaction of DC, NK and γδ T cells in the innate immune response is depicted, although clear interaction is thus far not completely understood. Ultimately, after FMDV infection and replication, systemic IL-10 produced by cDCs is detected, thus directing the adaptive immune response toward a stronger humoral stimulation rather than a T-cell mediated response.

The interaction of FMDV with the host begins via infection of epithelial cells of the nasopharyngeal mucosa in cattle (Arzt et al., 2010; Stenfeldt et al., 2015) or similar epithelium within crypts of oropharyngeal tonsils in pigs (Stenfeldt et al., 2014) [see previous section for more details]. After the primary infection, the virus may get in contact with NK cells, γδ T cells or APCs, either as a result of lytic infection of epithelial cells and subsequent phagocytosis (Rigden et al., 2002) and/or lytic action on damaged infected tissue (Toka et al., 2009) (Figure 3). Furthermore, immune cells such as Mφ (McCullough et al., 1988; Mason et al., 2003) or DCs can be infected in an antibody-mediated internalization process, thus facilitating cell contact during infection (Díaz-San Segundo et al., 2006; Guzylack-Piriou et al., 2006).

Similarly to other species, porcine NK cells are identified as CD2+/CD8+/CD3- cells (Denyer et al., 2006). At rest, these cells show minimal cytolytic activity even toward FMDV infected epithelial cells. However, cytotoxicity and expression of IFNγ expression significantly increased upon stimulation with cytokines such as IL-2, IL-12, IL-15, IL-18, or IFNα (Pintarič et al., 2008; Toka et al., 2009), including cytotoxicity against FMDV infected target cells. However, shortly after FMDV infection in swine, the number of circulating NK cells transiently decrease. Moreover, NK cells isolated from FMDV-infected swine are dysfunctional due to the lack of IFNγ secretion and the inability to kill NK-sensitive targets such as K562, a human lymphocytic cell line, or porcine fibroblasts, infected with FMDV (Toka et al., 2009). The same authors have proposed several mechanisms to explain the defective behavior of NKs during FMDV infection in swine. Induction of TLR3 and SOCS3 mRNAs may negatively affect the expression of IFNα, which has shown to be required for NK activation. Additionally, NK unresponsiveness may be caused by a reduction in the levels of cytokines IL-12, IL-15, and IL-18, upon viral infection. Interestingly, it has been shown that the expression of NK surface receptors, NKG2D, NKp80, and cytoplasmic granzyme B, all key molecules involved in cellular activation and inhibition, is minimally affected in infected swine (Toka et al., 2009). Similarly to swine, bovine NK cells appear to have a CD2+/-/CD8+/-/CD3- phenotype, express natural cytotoxicity receptors like CD335 (Boysen et al., 2006b; Bastos et al., 2008) and are capable of lysing infected target cells (Boysen et al., 2006a). However, NK cells originated in FMDV infected cows have an elevated cytotoxic-function against bovine target epithelial cells *in vitro* (Patch et al., 2014). The ability of cattle NK cells of responding to FMDV infection represents a difference in pathogenesis between cattle and pigs. These data may partially explain the higher morbidity of FMD in outbreak situations in swine as compared to cattle, due to significantly higher levels of virus shedding in this species (Donaldson et al., 1970), however, more studies to understand the importance of NK response during FMDV infection in cattle are needed.

At least 12 different γδ T cell populations have been found in the thymus of pigs, based on the expression of CD1, CD2, CD4, CD8, and CD45RC (Šinkora et al., 2005). Interestingly, *in vitro* exposure of naïve γδ T cells to high concentrations of inactivated FMDV vaccine antigen can induce the expression of many cytokines and chemokines (Takamatsu et al., 2006). However, further studies are needed to elucidate the role of each γδ T cell swine population in modulating the innate and adaptive immune responses to FMDV infection and vaccination.

On the other hand, the landscape of these cells during FMDV infection in cattle is better characterized. There are two major populations of γδ T cells in cattle whose differentiation is based on the expression of the protein WC1, a cysteine-rich scavenger receptor. Bovine γδ T cells are WC1+/CD3+/CD5+/CD2-/CD6-/CD8- or WC1-/CD3+/CD5+/CD2+/CD6+/CD8+ (Wijngaard et al., 1992; Aruffo et al., 1997). Earlier reports demonstrated that γδ T cells from vaccinated cattle could respond to FMDV antigen (Amadori et al., 1995). More recently, examination of bovine γδ T cells during FMDV infection revealed that WC1+ γδ T cells show a transient activated phenotype characterized by upregulation of CD25, downregulation of CD62L and CD45RO, and increased expression of IFNγ (Toka et al., 2009). Interestingly, it has been shown that WC1+ γδ T cells acquire NK-like capabilities to kill target cells *in vitro* by increasing the expression of CD335 and perforin. Parallel to those changes, WC1+ γδ T cells also showed upregulation of MHC-II and CD13, suggesting that after exposure to FMDV *in vivo* they may also act as APCs, as previously suggested (Collins et al., 1998). However, more studies are needed to determine whether the changes observed in γδ T cells are the result of direct virus-cell interactions or a bystander consequence, and how FMDV modulates their activity considering the prevalence of this specific type of T-cells in cattle (Wijngaard et al., 1992).

The DCs can be broadly classified into two lineage populations: pDC, specializing in the production of cytokines, most notably types I and III IFN, and conventional DCs (cDCs), which are potent APCs (Swiecki and Colonna, 2015). cDCs have been defined as sentinel cells that capture, process and present antigen upon migration to lymphoid tissues, resulting in activation and proliferation of rare T cell clones, and linking innate and adaptive immunity (Steinman, 2008). Although it has been shown that the interactions between FMDV and APCs are mostly abortive because no virions are produced (Rigden et al., 2002; Guzylack-Piriou et al., 2006; Díaz-San Segundo et al., 2009), heparan sulfate mediated viral uptake resulted in transient FMDV replication in cDCs (Harwood et al., 2008). It is worth mentioning that DC functionality is affected upon FMDV infection. During acute infection, the virus stimulates swine and cattle cDCs to produce IL-10, a cytokine that directs the immune response toward a stronger humoral rather than a T-cell mediated adaptive response (Díaz-San Segundo et al., 2009; Sei et al., 2016). FMDV also blocks the ability of porcine DCs to differentiate into mature cDCs (Díaz-San Segundo et al., 2009) and impairs the response to stimulation by TLR ligands (Nfon et al., 2008). Similarly, during FDMV infection in cattle, the number of bovine CD11c- cDCs, but not CD11c+ cDCs, is significantly decreased during the peak of viremia, the expression of MHC-II molecules on all bovine cDC populations is dramatically downregulated and the processing of exogenous antigen is impaired (Sei et al., 2016).

Another important set of tissue resident DCs affected during FMDV infection are the LC, a particular subset of DCs that expresses langerin and is found in the epidermis (Clayton et al., 2017). FMDV can attach to, and become internalized by porcine LCs *in vitro*, although, no viral RNA replication or production of viral proteins upon internalization could be detected (Bautista et al., 2005). Furthermore, after *ex vivo* stimulation, IFNα production is impaired in LCs derived from FMDV-infected pigs, although the ability to present antigen remains intact (Nfon et al., 2008).

Porcine pDCs also sense FMDV through TLR7 and produce IFNα in response to infection, but the levels of secreted IFN are relatively modest when compared to other viruses such as influenza (Bel et al., 2011; Lannes et al., 2012). Furthermore, *in vivo* studies in swine have shown that during FMDV infection there is a depletion of pDCs in peripheral blood, and remaining pDCs produce less IFNα upon *ex vivo* stimulation with TLR ligands or virus (Nfon et al., 2010). In contrast, high levels of type I IFN are produced after *ex vivo* stimulation of bovine pDCs with TLR9 agonist CpG and FMDV immune complexes (Reid et al., 2011). Furthermore, a robust systemic *in vivo* type I IFN response is detected in cattle infected with FMDV (Stenfeldt et al., 2011; Perez-Martin et al., 2012) and the number of systemic mature bovine CD4+ MHC-II+ pDCs is increased during FMDV infection, while levels of MHC-II and immature CD4+ MHC-II- pDCs are declined (Sei et al., 2016).

Monocytes/Mφ are part of the innate response to viral infection and are essential for the rapid clearing of pathogens at the sites of infection. Similarly to what happens with pDCs, FMDV also enters Mφ by utilizing the FcγRII receptor in an antibody-dependent internalization manner (McCullough et al., 1988; Baxt and Mason, 1995). Interestingly, it has been reported that, FMDV infectivity endures at least for 10–24 h after viral uptake in the absence of productive infection of porcine Mφ, (Rigden et al., 2002). Implications of these observations favor a model in which Mφ act as transporters and disseminators of viable virions to distant sites of the body where the virus can infect and replicate in other cells. On the other hand, in cattle, CD14+ monocyte frequency increases following inoculation with FMDV (Sei et al., 2016), consistently with previous reports demonstrating that the number of blood monocytes augment following vaccination and challenge with FMDV (Sigal et al., 1992). In this case, blood monocytes may function as cDCs, in which the reduced levels of MHC-II compromise antigen presentation.

Foot-and-mouth disease virus infection also affects the innate immune response at the cytokine level in both, swine and cattle. *In vivo* cytokine profile analysis during the 1st week of infection shows a systemic decrease of pro-inflammatory cytokines (IL-1β, IL-6, and TNFα), while an increase of the anti-inflammatory cytokine IL-10 and IFNα is detected (Nfon et al., 2010; Diaz-San Segundo et al., 2012, 2013; Perez-Martin et al., 2012; Sei et al., 2016). Most likely, these changes are related with the early T cell unresponsiveness and lymphopenia described in swine and cattle during FMDV infection (Bautista et al., 2005; Díaz-San Segundo et al., 2006; Perez-Martin et al., 2012; Sei et al., 2016). Indeed, it has been reported that, IL-10, inhibits a broad spectrum of cellular responses, and causes immunosuppression and persistence *in vivo* for other viruses (Brooks et al., 2006). With respect to FMDV-induced lymphopenia, no clear mechanism has thus far been elucidated. Apoptosis mediated cell death has been ruled out in *ex vivo* infected swine and bovine lymphocytes, despite detection of productive FMDV replication (Díaz-San Segundo et al., 2006; Joshi et al., 2009). These results suggested that lymphopenia could be directly attributed to a cytolytic or alternative mechanism of cell death. Another possibility would be that the observed lymphopenia is the result of a transient egression of lymphocytes from blood to infected tissues, primarily influenced by an increase of systemic IFN. Support for this hypothesis comes from murine studies in which administration of IFNα *in vivo* resulted in broad lymphopenia which was not observed in IFNα/β receptor KO mice (Kamphuis et al., 2006). Furthermore, these results are consistent with studies in livestock species in which increased levels of type I IFNs were detected in blood of infected swine (Nfon et al., 2010) or cattle (Stenfeldt et al., 2011; Perez-Martin et al., 2012). However, overexpression of IFN using a replication defective human Adenovirus 5 vector (Ad5-IFN) did not induce lymphopenia during the peak of IFN detection in cattle (Perez-Martin et al., 2012). Further studies are needed to understand the mechanisms of FMDV induced lymphopenia and the role IFN might play in this clinical sign.

Despite the differences in response to FMDV between swine and cattle, it is clear that the virus triggers a state of immunosuppression in both species, favoring a Th2 cell/cytokine-like environment that induces a strong FMDV-specific neutralizing antibody response to ultimately clear the virus. In fact, the serological response of naïve pigs and cattle to FMDV infection is characterized by a rapid surge of anti-FMDV immunoglobulin M (IgM) that peaks approximately at 7 days succeeded by a sustained anti-FMDV IgG response which remains at high titers beyond 28 days (Juleff et al., 2009; Pacheco et al., 2010b).

### FMDV Is Remarkably Sensitive to IFN Treatment

Despite the robust IFN response detected during the peak of viremia in cattle, FMDV is very sensitive to IFN (Ahl and Rump, 1976; Diaz-San Segundo et al., 2013). In fact, it has been demonstrated that when IFN (either type I, type II, or type III IFNs) -mediated antiviral stage is induced prior to virus infection, replication of all seven FMDV serotypes can be dramatically inhibited (Chinsangaram et al., 1999, 2001; Moraes et al., 2007; Díaz-San Segundo et al., 2011; Grubman et al., 2012). Furthermore, animals inoculated with Ad5 vectors expressing type I or type III IFN are efficiently protected against challenge at 1 day post inoculation in swine (Moraes et al., 2003, 2007; Diaz-San Segundo et al., 2010; Dias et al., 2011) and in cattle (Wu et al., 2003; Perez-Martin et al., 2012). Interestingly, treatment of swine with Ad5-IFNα is effective against multiple FMDV serotypes and protection lasts for approximately 3–5 days (Moraes et al., 2003). A synergistic effect against FMD has been detected when types I and II IFNs were co-administered in swine (Moraes et al., 2007; Kim et al., 2014). Studies aimed at elucidating the mechanisms by which IFN protects swine against FMD have demonstrated that protection of swine inoculated with Ad5-IFNα correlates with a local recruitment of skin DCs (Diaz-San Segundo et al., 2010) with partial maturation, increased expression of CD80/86 and decreased phagocytic activity (Diaz-San Segundo et al., 2013).

In essence, combined delivery of FMDV vaccines and select immunomodulatory constructs, such as exogenous sources of IFN, could provide a rapid onset and broad protection to deter primary virus infection prior to the vaccine-induced antibody response. In a proof of concept study in swine, Moraes et al. (2003) treated animals with a combination of Ad5-poIFNα and Ad5-FMD subunit vaccine challenging at 5 dpi with FMDV A24. Treated animals were completely protected against FMD and developed a significant adaptive immune response (Moraes et al., 2003). More recently, similar results were described in cattle treated with a combination of Ad5-boIFN-λ3 and Ad5-O1Manisa (Ad5-O1M) against aerosol challenge with FMDV (Diaz-San Segundo et al., 2016).

## Concluding Remarks

In this review, we have summarized a wealth of information that over the years has advanced our understanding of the many components required for the FMDV life cycle and the host restriction mechanisms targeted by the virus to favor its replication. FMDV has evolved an unusually fast replication rate to conquer the host. The virus uses its entire genome to juggle with the cellular machinery involved in induction and modulation of innate immunity accomplishing severe morbidity soon after infection. The most efficient mechanism by which FMDV defeats the host antiviral response is perhaps the early shut-off on host translation, that limits the expression of antiviral molecules during the transition from local to systemic infection. However, the virus also uses many additional strategies to finely modulate and take advantage of the induced antiviral pathways gaining fitness and rapidly proliferating. FMDV successfully achieves these goals by: inhibiting RNA sensing by host PRRs; interfering with PTMs of many factors involved in IFN pathway activation and cellular trafficking; causing degradation of the transcription factors that govern expression of IFN, inflammatory cytokines and ISGs; modifying the chromatin architecture at the IFN/ISG loci; inducing rearrangements of cell membranes to optimize replication and limit protein secretion; disrupting formation of stress organelles such as SGs; modulating metabolic pathways such as autophagy and apoptosis, while simultaneously destabilizing pathways and components involved in stimulating the IFN pathway. Finally, FMDV uses all its proteins, structural and non-structural, to directly interact with most of the proteins that mediate the IFN auto/paracrine loops.

A relatively short replication cycle allows FMDV to quickly establish infection in local tissues and disseminate throughout the body of the animal host in less than a week. Interestingly, despite the local inhibition in the production of IFN in infected cells early post infection, significant levels of IFN protein can be detected systemically following kinetics of viremia. A rapid uptake of virus particles mediated by phagocytosis or Fc dependent internalization by APCs skews the adaptive response to the production of relatively high amounts of antibodies although surface expression of MHC class I and II molecules declines post infection. Furthermore, FMDV infection results in a rapid, but transient lymphopenia, reducing the number of circulating T and B cells and adversely affecting T cell function, although no clear mechanism has thus far been elucidated.

The theme for this successful pathogen appears to be the ability to counteract both, the host innate and adaptive immune response, at many levels. Uncovering additional FMDV host interactions will be instrumental in understanding in detail the molecular mechanisms by which FMDV counteracts the host immune response and target novel improved disease control strategies.

## Author Contributions

GM and TS planned, coordinated, and wrote the manuscript. FS drafted the sections on analysis of the host immune responses in vivo and contributed scientific comments to the manuscript. CS drafted the section on FMDV pathogenesis and contributed scientific comments to the whole manuscript. JA contributed scientific comments to the manuscript.

## Conflict of Interest Statement

The authors declare that the research was conducted in the absence of any commercial or financial relationships that could be construed as a potential conflict of interest.

## Abbreviations

ADNP: activity dependent neuroprotective protein
AP1: activator protein 1
APC: antigen presenting cell
ATF: activating transcription factor
ATG: autophagy-related gene
BHK: baby hamster kidney
CARD: caspase activation and recruitment domain
CCPs: clathrin-coated pits
CD: cluster of differentiation
cDCs: conventional dendritic cells
cGAS: cyclic GMP-AMP synthase
CXCL10: C-X-C Motif Chemokine Ligand 10
EHMT2: euchromatic histone-lysine *N*-methyltransferase 2
eIF: eukaryotic translation initiation factor
ER: endoplasmic reticulum
ERK: extracellular signal-regulated kinase
FMDV: foot-and-mouth disease virus
G3BP1/2: GTPase-activating protein binding protein ½
GBP1: guanylate-binding protein 1
HSPB1: heat shock protein B1
IκB: Inhibitor of κB kinases
IFN: interferon
IFNR: IFN receptor
IFP35: IFN induced protein 35
IL: interleukin
ILC: immune lymphoid cell
INDO: Indoleamine-pyrrole 2,3-dioxygenase
IRAK: interleukin-1 receptor-associated kinase
IRES: internal ribosome entry site
IRF: IFN regulatory factor
ISG: IFN stimulated gene
ISGF3G: IFN-stimulated transcription factor 3, gamma
ISRE IFN: stimulated response element
ITAF: IRES transacting factors
JAK: Janus kinase
KO: knockout
KPNA: nuclear localization signal receptor protein
LCs: Langerhans cells
LGP2: laboratory of genetics protein 2
LLV: leaderless virus
MAM: mitochondria associated membranes
MAPK: mitogen activated protein kinase
MAVS: mitochondrial antiviral signaling protein
MCP-1: monocyte chemoattractant protein 1
MDA5: melanoma differentiation associated genes
MHC: major histocompatibility complex
MIP3α: macrophage inflammatory protein-3 alpha
mRNP: messenger ribonucleoprotein
Mx-1: myxovirus resistant 1
MyD88: myeloid differentiation primary response protein 88d
Mφ: macrophage
NEMO: NF-κB essential modulator
NF-κB: nuclear factor-κB
NKC: natural killer cell
NLR: NOD leucine-rich repeat-containing receptors
NOD: nucleotide-binding oligomerization domain
OAS: oligoadenylate synthase proteins
ORF: open reading frame
PAMP: pathogen associated molecular patterns
PBMC: peripheral blood monocyte cell
PCBP2: poly(rC) binding protein 2
pDCs: plasmacytoid dendritic cells
PIAS: protein inhibitor of activated STAT
PK: pseudo knots
PKR: protein kinase R
PMC: peritoneal mast cell
poly-I:C: polyinosinic:polycytidylic acid
poly-Ub: polyubiquitination
PRR: pathogen recognition receptors
PTBP: polypyrimidine tract-binding protein
PTM: post-translational modifications
RANTES: regulated on activation; normal T cell expressed and secreted
RdRp: RNA-dependent RNA polymerase
RHA: RNA helicase A
RIG-I: retinoic acid-inducible gene I
RLR: RIG-I like receptor
SAP: SAF-ACINUS-PIAS
SeV: Sendai virus
SGs: stress granules
SOCS3: suppressor of cytokine signaling 3
STAT: signal transducer and transcription activator
TANK1: TRAF family member associated NF-κB activator 1
TBK: TANK binding kinase
TIR: toll/interleukin-1 receptor
TIRAP: toll/interleukin 1 receptor domain containing adaptor protein
TLR: Toll-like receptor
TNF: tumor necrosis factor
TRAF: TNF receptor associated factor
TRIF: TIR-domain-containing adapter-inducing interferon-β
TRIM: tripartite motif containing protein
TYK2: tyrosine kinase 2
UB: ubiquitin
UBL: ubiquitin-like
USP18: ubiquitin-specific protease 18
UTR: untranslated region
WC1: workshop cluster 1
WT: wild type
